# A microscopic Kapitza pendulum

**DOI:** 10.1038/s41598-018-31392-8

**Published:** 2018-08-30

**Authors:** Christopher J. Richards, Thomas J. Smart, Philip H. Jones, David Cubero

**Affiliations:** 1 Department of Physics & Astronomy, University College London, Gower Street, London, WC1E 6BT UK; 2 Departamento de Fisica Aplicada I, EPS, Universidad de Sevilla, Calle Virgen de África 7, 41011 Sevilla, Spain

## Abstract

Pyotr Kapitza studied in 1951 the unusual equilibrium features of a rigid pendulum when its point of suspension is under a high-frequency vertical vibration. A sufficiently fast vibration makes the top position stable, putting the pendulum in an inverted orientation that seemingly defies gravity. Kapitza’s analytical method, based on an asymptotic separation of fast and slow variables yielding a renormalized potential, has found application in many diverse areas. Here we study Kapitza’s pendulum going beyond its typical idealizations, by explicitly considering its finite stiffness and the dissipative interaction with the surrounding medium, and using similar theoretical methods as Kapitza. The pendulum is realized at the micrometre scale using a colloidal particle suspended in water and trapped by optical tweezers. Though the strong dissipation present at this scale prevents the inverted pendulum regime, new ones appear in which the equilibrium positions are displaced to the side, and with transitions between them determined either by the driving frequency or the friction coefficient. These new regimes could be exploited in applications aimed at particle separation at small scales.

## Introduction

Kapitza’s analysis^1,2^ of a vibrating pendulum arrived more than 40 years after the counter-intuitive phenomenon of inverted stability had been reported^3^. His celebrated analytical insight has found application in many fields across diverse length scales: from macroscopic applications^4–6^, where fast vibrations play an important role in the vibrational transport or manipulation of granular media, to microscopic and nanoscopic applications^7–20^, in areas such as Brownian motors^9,13,21^, atomic physics^8,10–13^, optical systems^14,15^ or biophysics^17–20^.

More specifically, to mention a few examples, it has been shown that the addition of a sufficiently high frequency force may be able to suppress the firing activity of a neuron^17^, the stochastic-resonance response in a quantum system^11^, enable the experimental realization of the Harper-Hofstadter Hamiltonian with cold atoms^8,12^ or suppress transport in a ratchet system^9,13^. Particle transport at submicron scales is of paramount interest in the design and operation of nanodevices^21^. In most cases inertial effects can be neglected at these scales: the particle’s mass *m* is very small, and the viscous coefficient *η* very large, yielding overdamped dynamics. Diffusion and other transport properties depend on *η*, which is related to the particle size, but are otherwise largely independent of *m*, hindering applications like particle separation with respect to the particle’s mass. The addition of a high frequency force has already been proposed^9^ in one-dimensional spacially periodic systems, as a tool to maintain inertial effects —via a dependency on *η*/*m*— useful for particle segregation purposes, in the presence of overdamped dynamics. In this regard, we report here similar benefits for high frequency forces in the circular system studied by Kapitza, i.e. theoretical dependency on the damping constant *η*/*m* despite strong overdamped dynamics. The analytical predictions are demonstrated both in numerical simulations and experimentally at the micrometer scale.

## Results

**Figure 1:**
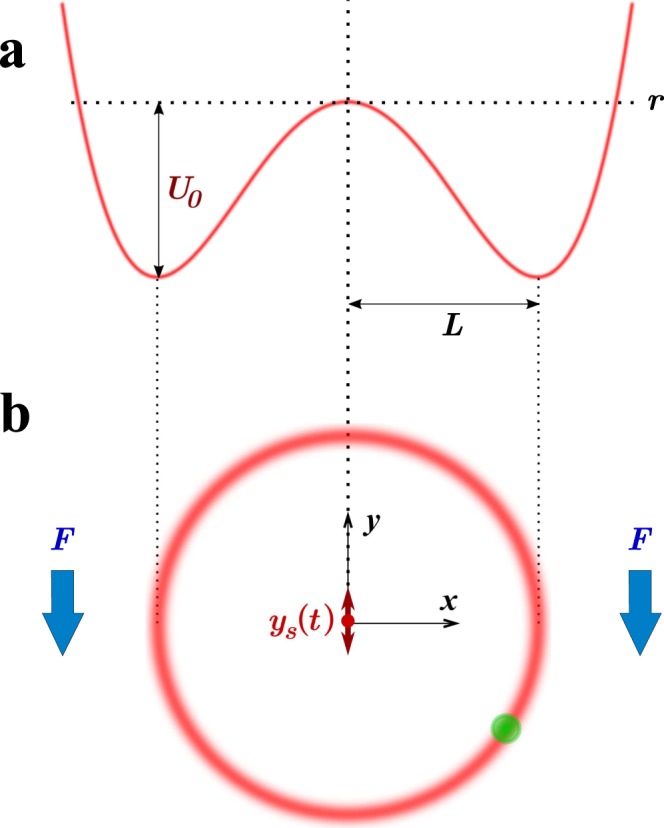
System setup. (**a**) Quartic pendulum potential *U*(*r*), Eq. (3), used in the analytical calculations in the very high-frequency limit. (**b**) Schematic figure showing the colloidal particle confined by the (optical) pendulum potential under the downward force *F*, which is created in the experiment by a uniform drag. The pendulum is subject to a driving, fast vibration *y*_*s*_(*t*), Eq. (2), in the *y*-direction.

We study the dynamical regimes of a vibrating rigid pendulum with a finite stiffness in the presence of dissipation. Experimentally this is realised using a microscopic particle suspended in water and confined with optical tweezers. As an analytical model, we consider a two-dimensional system with a single particle of mass *m* subject to the following equation of motion

1 $$\begin{array}{c}m\ddot{x}=-\,m\gamma \dot{x}-\frac{\partial U}{\partial x}(x,y-{y}_{s})+{\xi }_{x}\\ m\ddot{y}=-\,m\gamma \dot{y}-\frac{\partial U}{\partial y}(x,y-{y}_{s})-F+{\xi }_{y},\end{array}$$

where *η* = *mγ* is the fluid drag coefficient (i.e. *γ* is the viscous drag coefficient normalised by the particle mass), *U*(*x*, *y*) is the pendulum potential, and *F* is the pendulum downward force—e.g. weight in the case of the classical Kapitza pendulum. The high-frequency vibration of the pendulum suspension is applied parallel to the direction of the force *F*, as shown in Fig. 1(b), and is described by

2 $${y}_{s}(t)=-\,a\,\cos (\omega t+{\phi }_{0}),$$

with *a*, *ω* and *φ*_0_ being the driving constants amplitude, frequency and phase respectively. In Eq. (1) the force terms (*ξ*_*x*_, *ξ*_*y*_) are multivariate Gaussian white noise accounting for the fluctuating interaction of the colloidal particle with the surrounding fluid, with properties $\langle {\xi }_{x}\rangle =\langle {\xi }_{y}\rangle =0$, $\langle {\xi }_{i}(t){\xi }_{j}(t^{\prime} )\rangle =\mathrm{(2}m\gamma {k}_{B}T)\delta (t-t^{\prime} ){\delta }_{i,j}$, *i*, *j* = *x*, *y*, where *T* is the fluid temperature^22^. Albeit relevant to the full description of our experiments, this random force does not alter the analysis presented here, and for the sake of simplicity will be omitted in the following. The model system described by (1)–(2) can be equally used to study a macroscopic pendulum immersed in a viscous fluid, although at this scale the fluctuating forces *ξ*_*i*_ will be negligible.

As a concrete example we consider the following quartic potential, illustrated in Fig. 1,

3 $$U(x,y)=({x}^{2}+{y}^{2})({x}^{2}+{y}^{2}-2{L}^{2}){U}_{0}/{L}^{4}={r}^{2}({r}^{2}-2{L}^{2}){U}_{0}/{L}^{4},$$

because it is a simple form that captures the main physics of a mathematical pendulum of length *L* for sufficiently large *U*_0_ > 0, and in addition is amenable to analytical calculations. For small deviations around the minimum, the potential can be expanded as $U(x,y)\approx -{U}_{0}+m{\omega }_{0}^{2}{(\sqrt{{x}^{2}+{y}^{2}}-L)}^{2}/2$, where ${\omega }_{0}={(8{U}_{0}/m{L}^{2})}^{\mathrm{1/2}}$ is the pendulum’s proper frequency. Formally, it becomes completely rigid in the limit *ω*_0_ → ∞. Throughout the paper, we will assume ${\omega }_{0}^{2}\gg F/mL={{\rm{\Omega }}}^{2}$ (with Ω the pendulum oscillation frequency) in order to maintain pendulum-like properties. In the limit of high rigidity, the specific details of the potential (3) —other than the proper frequency *ω*_0_— become irrelevant, and the results are valid for any physically acceptable pendulum potential.

### The very high-frequency limit

Let us now study the effect of a vibration with an associated frequency *ω* which is much larger than any characteristic frequency of the system. This is equivalent to formally taking the asymptotic limit *ω* → ∞ while keeping other parameters constant. Following Kapitza’s celebrated analysis, it is expected that *x*(*t*) and *y*(*t*) change on a much slower time-scale than that of the high-frequency drive, thus the explicit time dependence in the equation of motion (1) can be removed by integrating over a time interval that includes many high-frequency periods but in which *x*(*t*) and *y*(*t*) do not appreciably change. Equivalently, this explicit fast dependence can also be eliminated by noting that *x*(*t*) and *y*(*t*) should be insensitive to the driving phase *φ*_0_. Integrating Eq. (1) over *φ*_0_ yields the following equations

4 $$\begin{array}{c}m\ddot{x}=-\,m\gamma \dot{x}-\frac{\partial {U}_{{\rm{eff}}}}{\partial x}\\ m\ddot{y}=-\,m\gamma \dot{y}-\frac{\partial {U}_{{\rm{eff}}}}{\partial y}-F,\end{array}$$

where *U*_eff_ is an effective potential given by:

5 $${U}_{{\rm{eff}}}(x,y)=U(x,y)+\frac{1}{8}m{\omega }_{0}^{2}{(\frac{a}{L})}^{2}({x}^{2}+3{y}^{2}).$$

This approximation can be also obtained as the second order of a multiple time-scale expansion^23^ —for non-dissipative systems, like the quantum two-level system of ref.^24^, it is only valid up to a given time-scale (second order), with corrections needed for coarser time-scales (third order), though in the dissipative case it is usually valid at higher orders due to the presence of attractors. Furthermore, note also that the leading order correction term considered in section 30 of the book^5^ vanishes in this case, but the next order correction leads precisely to the equations presented here, Eqs (4) and (5).

With the effective potential (5), the bottom of the pendulum, (*x*, *y*) = (0,−*L*), is still stable for driving amplitudes in the range $0\le a < {a}_{1}$, where the threshold *a*_1_ can be computed to lowest order in ${\rm{\Omega }}/{\omega }_{0}\ll 1$ as

6 $$\frac{{a}_{1}}{L}\approx \sqrt{2}\frac{{\rm{\Omega }}}{{\omega }_{0}}$$

In the interval *a*_1_ < *a* < *a*_2_, with ${a}_{2}\approx \sqrt{2}L$ (also to the lowest order in Ω/*ω*_0_), there are only the following stable equilibrium positions

7 $$\begin{array}{c}x=\pm L\sqrt{1-{(a/L)}^{2}\mathrm{/2}-\mathrm{4(}{\rm{\Omega }}/{\omega }_{0}{)}^{4}/(a/L{)}^{4}},\\ y=-\,2L{({\rm{\Omega }}/{\omega }_{0})}^{2}/{(a/L)}^{2}.\end{array}$$

For *a*_2_ < *a* there is only one equilibrium point, located slightly below the pendulum’s center, near (*x*, *y*) = (0, 0).

Notice that none of these equilibrium points are associated with the inverted pendulum. For that, a very rigid pendulum is needed, that is, with a pendulum frequency *ω*_0_ much larger than the driving frequency *ω*.

### A rigid pendulum with friction

In the presence of friction, the necessary condition $\omega \ll {\omega }_{0}$ is not sufficient to observe the inverted pendulum effect, i.e. the stabilization of the pendulum’s top equilibrium position. In order to understand this, let us consider the effect of increasing the friction coefficient *γ* while keeping all other parameters fixed. Since the friction force opposes the particle’s movement, as *γ* is increased the particle is increasingly slowed down, taking longer to reach the equilibrium positions. Thus, the friction slows down the system response, effectively decreasing its characteristic frequencies. For large enough friction the system’s response can be so slow, much slower than the drive of frequency *ω*, that it is effectively carried into the very high-frequency regime discussed above, regardless of whether $\omega \ll {\omega }_{0}$ is satisfied or not.

Indeed, in the overdamped regime the friction is so large that the inertial terms $m(\ddot{x},\ddot{y})$ in (1) can be neglected in comparison with the friction force $-m\gamma (\dot{x},\dot{y})$, and the equation of motion thus becomes first order in time. In this regime the characteristic timescale for relaxation of the pendulum length, *τ*_0_, is given by the ratio of friction to the pendulum stiffness, thus ${\tau }_{0}^{-1}={\omega }_{0}^{2}/\gamma$. Since this is the largest intrinsic frequency in the system, the condition for a (very) high-frequency driving is thus ${\omega }_{0}^{2}/\gamma \ll \omega$, in contrast with $\omega \ll {\omega }_{0}$ which is needed in the weakly damped regime. A large enough friction can thus put the system into the very high-frequency regime.

In order to go beyond the very high-frequency regime analytically, let us consider the asymptotic limit *ω*_0_ → ∞, while keeping *γ*/*ω*_0_ constant (a constant that can be zero), so as to include the weakly damped regime. In practice, this asymptotic limit implies $\omega \ll {\omega }_{0}$ and thus also $\omega \gamma \ll {\omega }_{0}^{2}$. First, it is convenient to change into polar coordinates defined relative to the pendulum suspension point:

8 $$\begin{array}{c}x=r\,\sin \,\varphi \\ y=-\,r\,\cos \,\varphi +{y}_{s}.\end{array}$$

In these variables, the equations of motion (1) are written as

9 $$m(r\ddot{\varphi }+2\dot{r}\dot{\varphi }+{\ddot{y}}_{s}\,\sin \,\varphi )=-m\gamma (r\dot{\varphi }+{\dot{y}}_{s}\,\sin \,\varphi )-F\,\sin \,\varphi ,$$

10 $$\begin{array}{rcl}m(\ddot{r}-r{\dot{\varphi }}^{2}-{\ddot{y}}_{s}\,\cos \,\varphi ) & = & -m\gamma (\dot{r}-{\dot{y}}_{s}\,\cos \,\varphi )+F\,\cos \,\varphi \\  &  & -m{\omega }_{0}^{2}r({r}^{2}-{L}^{2})/2{L}^{2}.\end{array}$$

Since $({\rm{\Omega }}/{\omega }_{0})\ll 1$, *r* varies in a timescale much faster than that of *ϕ* or *y*_*s*_, and we can approximate the potential term in (10) by its linear form,

11 $$-m{\omega }_{0}^{2}r({r}^{2}-{L}^{2})/2{L}^{2}\sim -m{\omega }_{0}^{2}(r-L).$$

Then, the dynamics of *r*(*t*) will be either fast oscillations (weakly damped) around its *equilibrium* position *r*_0_, or a rapid decay (overdamped) to *r*_0_. Since *r*_0_(*t*) varies in a timescale much slower than that of *r*(*t*), it can be easily calculated from (10)–(11) in the leading orders as

12 $${r}_{0}\sim L+\frac{{\ddot{y}}_{s}\,\cos \,\varphi +\gamma {\dot{y}}_{s}\,\cos \,\varphi }{{\omega }_{0}^{2}}.$$

Inserting $r(t)\sim {r}_{0}$ into (9), and neglecting the terms that vanish in the limit *ω*_0_ → ∞, *γ*/*ω*_0_ =  constant, yields

13 $$\begin{array}{rcl}mL\ddot{\varphi } & = & -m\gamma (L\dot{\varphi }+{\dot{y}}_{s}{\rm{\sin }}\varphi )-(F+m{\ddot{y}}_{s}){\rm{\sin }}\varphi \\  &  & -m{(\frac{\gamma }{{\omega }_{0}})}^{2}{\dot{y}}_{s}\dot{\varphi }\,{\rm{\cos }}\varphi \mathrm{.}\end{array}$$

This equation generalizes Kapitza’s original equation^1^ for the case of moderate friction coefficients, being valid in a range that goes from zero to values holding $\gamma \ll {\omega }_{0}^{2}/\omega$. Although this range excludes values such that ${\omega }_{0}^{2}/\omega \sim \gamma$, the regime of extremely large frictions ${\omega }_{0}^{2}/\omega \ll \gamma$ is already covered by the very high-frequency regime as discussed above. Note also that since every physically relevant pendulum potential can be aproximated by a linear form when its intrinsic frequency *ω*_0_ is large enough, (13) is valid for any pendulum potential in the stated limit.

Previous studies^25–27^ of Kapitza’s pendulum in the presence of dissipation considered an equation of motion which does not include the last term of Eq. (13), as expected after an infinitely-rigid pendulum assumption (*ω*_0_ = ∞), but also ignore the term proportional to ${\dot{y}}_{s}\,\sin \,\varphi$ in (13), which accounts for the vertical vibrations of the point of suspension. This latter term is a consequence of the fact that the viscous force is proportional to the velocity of the pendulum relative to the fluid —it is absent in the study of ref.^27^. because the relevant damping force does not come from the surrounding fluid, but from a magnetic braking system specifically designed for the experiment, and thus proportional to the angular velocity of the pendulum only.

We now take equation (13) as a starting point for the (relatively) high-frequency limit *ω*→∞ with *aω* = constant. Following Kapitza, first we extract the rapid oscillations produced by the high-frequency drive by defining a slow variable $\tilde{\varphi }$ such as $\varphi =\tilde{\varphi }+\xi$, where $\xi =-\,({y}_{s}/L)\sin \,\tilde{\varphi }$. Expanding the sines and cosines in (13) for small *ξ* (note that the formal limit implies *a*→0, which in practice implies $a/L\ll 1$), $\sin \,\varphi \sim \,\sin \,\tilde{\varphi }+(\cos \,\tilde{\varphi })\xi$, $\cos \,\varphi \sim \,\cos \,\tilde{\varphi }-(\sin \,\tilde{\varphi })\xi$, and averaging over the driving phase *φ*_0_ yields the following equation for the slow variable

14 $$m\ddot{\tilde{\varphi }}=-m\gamma \dot{\tilde{\varphi }}-\,\frac{d{V}_{{\rm{eff}}}}{d\tilde{\varphi }},$$

where the new effective potential is given by

15 $${V}_{{\rm{eff}}}(\tilde{\varphi })=-\,m{{\rm{\Omega }}}^{2}[\cos \,\tilde{\varphi }+\frac{\lambda }{4}\,\cos \,2\tilde{\varphi }],$$

and

16 $$\lambda =\frac{1}{2}{(\frac{a}{L})}^{2}{(\frac{\omega }{{\rm{\Omega }}})}^{2}(1-\frac{{\gamma }^{2}}{{\omega }_{0}^{2}}).$$

The effective potential *V*_eff_ shows three distinct regimes depending on the value of *γ*. We find the inverted pendulum effect, i.e. a stable equilibrium point at the top of the pendulum $\tilde{\varphi }=\pi$, for frictions in the range $0 < \gamma  < {\gamma }_{1}$, where

17 $${\gamma }_{1}={\omega }_{0}\sqrt{1-2{(\frac{{\rm{\Omega }}/\omega }{a/L})}^{2}}.$$

Obviously, for this solution to exist, it is required that ${(a/L)}^{2} > \mathrm{2(}{\rm{\Omega }}/\omega {)}^{2}$. Thus, a necessary condition for the appearance of the Kaptiza effect is $\gamma  < {\omega }_{0}$. In the interval ${\gamma }_{1} < \gamma  < {\gamma }_{2}$, with

18 $${\gamma }_{2}={\omega }_{0}\sqrt{1+2{(\frac{{\rm{\Omega }}/\omega }{a/L})}^{2}},$$

the only stable equilibrium point is at the bottom of the pendulum, as for a normal pendulum that is unperturbed. However, by ${\gamma }_{2} < \gamma$ there has been a bifurcation of this bottom solution into the equilibrium points $\tilde{\varphi }=\pm \,{\varphi }_{1}$, where

19 $${\varphi }_{1}=\arccos (\,-\,\mathrm{1/}\lambda ).$$

The necessary condition ${\gamma }_{2} < \gamma$ for these latter solutions can be expressed as ${(a/L)}^{2} > \mathrm{2(}{\rm{\Omega }}/\omega {)}^{2}{({\gamma }^{2}/{\omega }_{0}^{2}-\mathrm{1)}}^{-1}$. They are reminiscent of the solutions (7) in the very high-frequency limit, which indeed can be written as

20 $${\varphi }_{1}^{{\rm{HF}}}=\arccos \,[\frac{\mathrm{2(}{\rm{\Omega }}/{\omega }_{0}{)}^{2}}{{(a/L)}^{2}\sqrt{1-\frac{1}{2}{(a/L)}^{2}}}].$$

However, in the case (19), valid for $\gamma \ll {\omega }_{0}^{2}/\omega$, the equilibrium angles vary continuously with the friction coefficient and driving frequency, while in the very high-frequency limit (${\omega }_{0}^{2}/\omega \ll \gamma$) they are independent of them.

**Figure 2:**
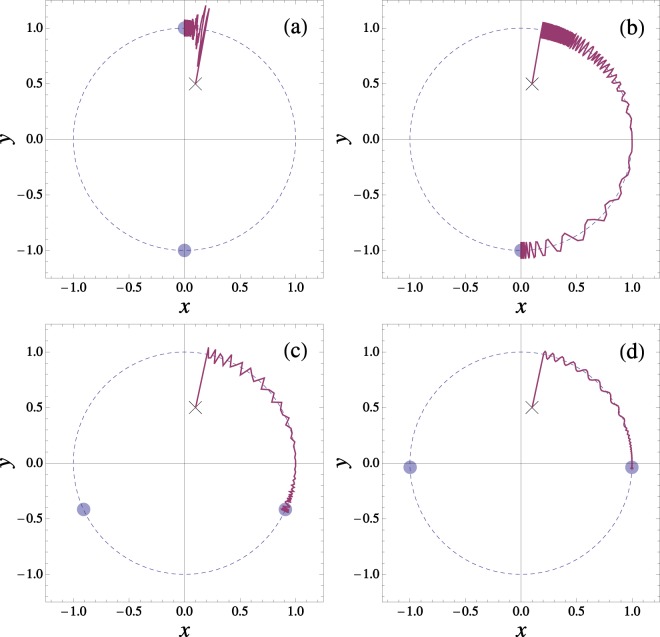
Simulations illustrating the dynamical regimes. Units are defined such that *L* = *m* = *F* = 1. The panels depict trajectories from the initial condition (*x*_0_,*y*_0_) = (0.1,0.5) (indicated with a cross) showing the dependence of the equilibrium position with the friction coefficient value. The pendulum frequency is *ω*_0_ = 100 and the driving parameters are *ω* = 20 and $a=(\sqrt{2}+\mathrm{0.05)/}\omega =0.0732$. The small disks in each panel indicate the position of the equilibrium points given by the analytical predictions. The friction coefficients are: (**a**) *γ* = 1 (inside inverted pendulum regime, defined for $0 < \gamma  < {\gamma }_{1}$), (**b**) *γ* = 30 (normal pendulum behavior, ${\gamma }_{1} < \gamma  < {\gamma }_{2}$), (**c**) *γ* = 180 (friction-filter regime, ${\gamma }_{2} < \gamma \ll {\omega }_{0}^{2}/\omega$), (**d**) *γ* = 1000 (very high-frequency regime, ${\omega }_{0}^{2}/\omega \ll \gamma$). Thresholds are predicted at *γ*_1_ ≈ 26, *γ*_2_ ≈ 139, and the reference value ${\omega }_{0}^{2}/\omega =500$.

**Figure 3:**
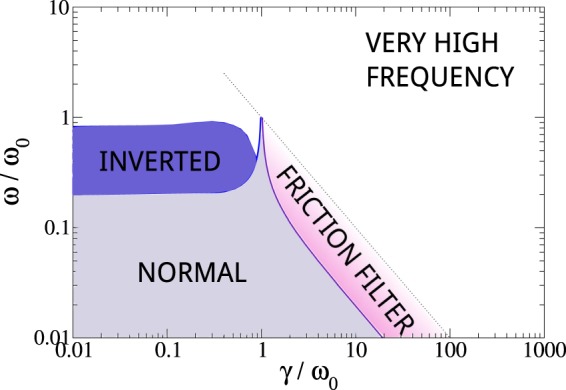
Diagram showing the different regimes under the same conditions as in Fig. 2, with *ω*_0_/Ω = 100 and *a*/*L* = 0.0732.

All these regimes can be explored by varying the friction coefficient while keeping all other parameters fixed, as illustrated in Fig. 2. Note that at each regime there is a distinct equilibrium point that could be used to discriminate between different friction values. Alternatively, the driving frequency can be used to control the dynamical regime, as shown in Fig. 3.

In order to test these predictions, we realized a microscopic pendulum using an optical tweezers experiment^28^ consisting of a *d* = 1.5 *μ*m diameter silica sphere, suspended in water and confined in a ring-shaped optical potential well with radius *L* of about 8 *μ*m. The optical trap was created by rapidly scanning the waist of a tightly focused laser beam around the circumference of a circle using galvanometer mirrors^29^. With sufficiently high laser intensity, confinement of the particle is such that the system behaves as a microscopic quasi-rigid pendulum in the overdamped regime (here ${\omega }_{0}\ll \gamma$, see Methods). The pendulum uniform force *F* was a viscous drag created by translating the suspending fluid in the plane of the pendulum at constant speed *v*_*s*_ using a piezo-stage, i.e. *F* = *mγv*_*s*_, in effect making gravity a tunable parameter in the experiment. It was then vibrated by superimposing an oscillation onto the position of the trap in a direction parallel to the drag force.

**Figure 4:**
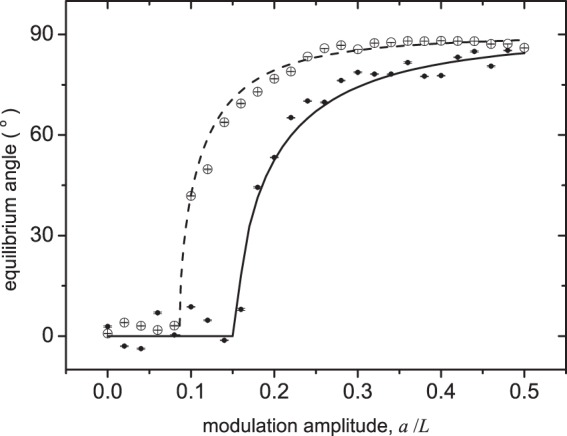
Experimental equilibrium angles as a function of the vibration amplitude in the very high-frequency regime for an optical pendulum with length *L* = 8.23 *μ*m. The vibration frequency was *ω* = 2*π* × 500 H*z*. Filled points correspond to an optical pedulum with proper frequency *ω*_0_ = 2*π* × 947 H*z* and stage speed *v*_*s*_ = 1 *μ*s^−1^ —leading to Ω = 2*π* × 105 H*z* and *γ*/*ω*_0_ = 598. Open points correspond to an experimental setup with an increased laser power, *ω*_0_ = 2*π* × 1490 H*z* (*γ*/*ω*_0_ = 380), and a reduced stage speed, such that Ω = 2*π* × 91 H*z*. The lines are the analytical predictions of Eq. (7), with a friction value calculated assuming Stokes’ law *γ* = 3*πζd*/*m*, where *ζ* is the dynamic viscosity of water.

Figure 4 shows the experimental results in the very high-frequency regime (${\omega }_{0}^{2}/\omega \ll \gamma$). To demonstrate the control over the pendulum dynamics in this regime, two experimental set-ups, with different laser power and stage speed, were considered. The parameters *L* and *ω*_0_ of the optical pendulum are measured from an experiment with no driving vibration (see Methods), and there are no fitted parameters. The quantitative agreement between the analytical prediction and the experimental data shown in Fig. 4 is very good, especially taking into account that the experimental pendulum potential does not have the quartic form of Eq. (3) among the space interval *a* spanned by the vibration—see section Methods.

**Figure 5:**
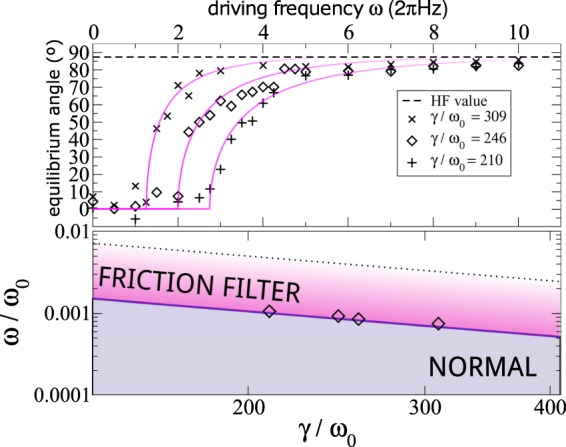
Experimental results (symbols) in the normal and friction-filter regime, with *a*/*L* = 0.25. The stage speed and laser power were varied to generate four sets of data, with varying values of *γ*/*ω*_0_, but with a common ratio *ω*_0_/Ω = 26.4. Top panel shows the equilibrium angle vs. *ω* for three of these sets. Solid lines indicate the friction-filter-regime prediction (19), and the horizontal dashed line the very high-frequency prediction (20). The diamonds in the bottom panel show the threshold from normal to friction-filter regime, estimated from the experimental data by assuming a regime transition when the angle crosses 20°. The solid line in the bottom panel is the threshold prediction given by (18).

With a change of experimental parameters we are also able to enter the regime ${\gamma }_{2} < \gamma \ll {\omega }_{0}^{2}/\omega$, the *friction-filter* regime illustrated in Figs 2(c) and 3. Figure 5 shows the experimental results with system parameters within the normal and friction-filter regime. The experimental equilibrium angles are well described by the analytical prediction of equation (19) —formally valid till $\omega \ll {\omega }_{0}^{2}/\gamma$— including the threshold (18) for stabilization away from $\tilde{\varphi }=0$ (the normal regime). The top panel of Fig. 5 also shows convergence of all curves towards the very high frequency value (20) (valid for ${\omega }_{0}^{2}/\gamma \ll \omega$).

Colloidal particles suspended in water exhibit a strongly overdamped dynamics, not permitting the observation of the inverted pendulum regime. The necesary condition for this regime, $\gamma  < {\omega }_{0}$ (see Eq. (17)), could be satisfied at the microscale for a similar experimental setup, but with air^30^ (see also Methods) as medium of suspension. However, the new regimes exhibit novel features with promising potential applications.

In the friction-filter regime, particles of different size have different friction coefficients, yielding different equilibrium angles. This fact could be used to sort small colloidal particles according to their size or refractive index, adding a possibility—or a new degree of fredoom for a combined approach—to existing techniques based on extended optical landscapes^31^ or Brownian ratchets^21,32–34^.

Finally, let us note that with the experimental setup considered here, the ratio ${\gamma }^{2}/({{\rm{\Omega }}}^{2}{\omega }_{0}^{2})=\eta L/({v}_{s}{\kappa }_{r})$, where ${\kappa }_{r}=m{\omega }_{0}^{2}$ is the optical pendulum stiffness, is independent of the particle’s mass *m* if the parameters *η*, *L* and *κ*_*r*_ are kept fixed. Thus, the equilibrium angles in the friction-filter regime (19) would be also be mass independent, in the overdamped regime ($\gamma /{\omega }_{0}\gg 1$) under these conditions, ignoring the mass sensitivity embedded in *γ* in the friction-filter regime. Two particles with same size (same *η*) and under the same optical potential, but with different mass, would be hard to distinguish in this experiment. In order to exploit, for particle segregation purposes, the residual inertial sensibility induced by the high frequency driving in this strongly overdamped system, it would suffice to replace the viscous drag force *F* = *mγv*_*s*_ with the particle’s own weight *F* = *mg*, where *g* is the acceleration of gravity, like in Kapizta’s original setup. Then the pendulum frequency Ω would become independent of *m*, and the angles (19) sensitive to *m*. This gravitational force is of the same order of magnitude than the drag force considered here. The use of a tunable *F*, on the other hand, has allowed us to explore experimentally the transition line between the normal and friction-filter regime, as shown in Fig. 5.

## Conclusions and outlook

In short, we have revisited Kapitza’s pendulum by explicitly considering a finite stiffness and viscous friction, which give rise to new regimes. The new analytical results are validated with numerical simulations and demonstrated at the micrometer scale with colloidal particles confined by optical tweezers. In the limit of a very high frequency vibration, i.e. faster than the pendulum proper frequency, the top point is not stable. The strong dissipation existing for colloidal particles in water prevents the observation of the inverted pendulum regime in the reported experiments. In contrast, the new dynamical regimes are all demonstrated experimentally. They are rich, allowing an increased control at the micrometer scale by means of the driving parameters or the friction coefficient.

The work presented here demonstrates the usefulness of the regime intermediate between static potentials, and those modulated at very high frequency, i.e. the *friction-filter* regime of the above. Modulation of the potential at these intermediate frequencies gives rise to dynamics that have a sensitivity to additional parameters, i.e. the friction coefficient, that may be usefully exploited in separating particles.

## Methods

### Experimental set-up

**Figure 6:**
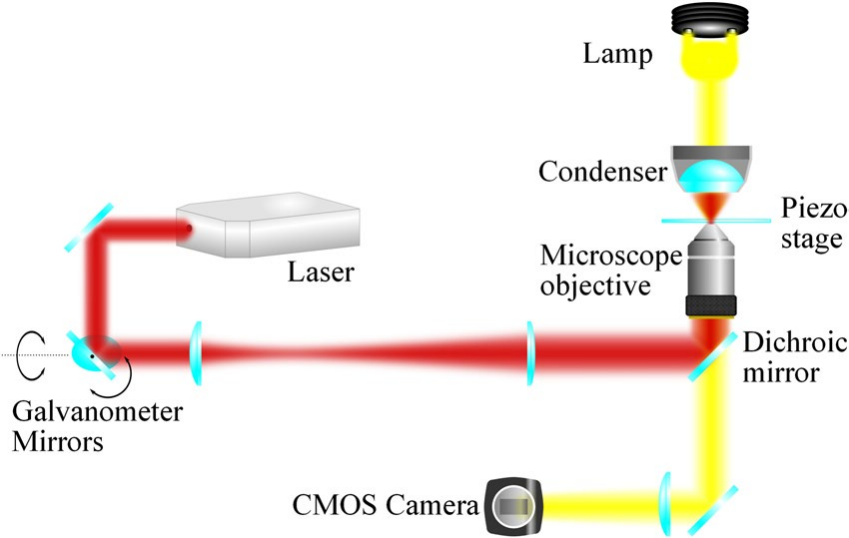
Schematic diagram of experimental apparatus. The galvanometer mirrors are mounted in a plane conjugate to the back aperture of the objective and steer the position of the beam waist in two dimensions in the trapping plane perpendicular to the propagation direction.

The optical tweezers is built around an inverted fluorescence microscope using a × 100, NA = 1.3 oil immersion objective lens. The microscope is also equipped with a three-axis nanopositioning piezo stage which we use to apply a controlled fluid drag to the trapped particle. The trapping laser beam is derived from a single mode Nd:YAG laser with maximum output power of 3 W. The path of the trapping beam includes a pair of orthogonally mounted galvanometer scanning mirrors^29^ in a plane made conjugate to the back aperture of the objective by a pair of lenses which also serve to increase the beam diameter to slightly overfill the aperture. The galvanometer mirrors can steer the location of the beam waist in two dimensions in the focal plane of the objective. Control of the scanning mirrors, and thus of the focal spot position, is performed via a National Instruments interface controlled by Matlab. The experiment is visualised using a CMOS camera. A schematic of the experimental apparatus is illustrated in Fig. 6.

The pendulum potential is created by scanning the laser beam waist in a circle at high frequency *f* = 1.2 kHz, such that a trapped particle experiences a time-averaged potential approximating a quasi-rigid pendulum. A particle trapped in the circular potential is subject to a uniform viscous drag force by moving the nanopositioning stage at a speed *v*_*s*_ of a few microns per second. The center of the optical potential is oscillated, in the direction parallel to the stage movement, by adding a small amplitude sinusoidal oscillation at angular frequency *ω* to one of the galvanometer mirrors.

### Video tracking

**Figure 7:**
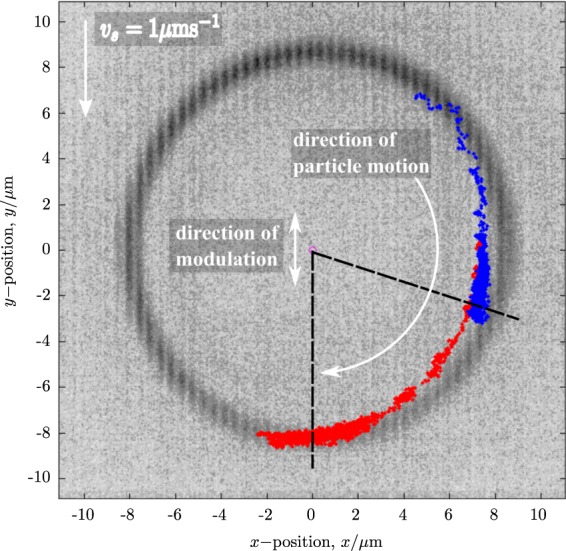
Video tracking output showing the trajectory of a colloidal particle in the optical pendulum potential with no driving (red symbols) and high-frequency stabilization with *a*/*L* = 0.3 (blue symbols). The predicted equilibrium angles of both situations are indicated by black dashed lines. A background image of the fast scanning laser spot (dark grey) illustrates the pendulum potential. Both the direction and speed of microscope stage movement—and hence direction of the drag force—and the direction of the pendulum vibration are indicated.

The position of the particle in the pendulum potential is tracked using digital video microscopy (DVM)^28^. Video is recorded at frame rates of up to 100 fps using the CMOS camera for up to 40 s over an area of approximately 22 *μ*m × 22 *μ*m. A custom-written (in Matlab) particle tracking algorithm is used to track the position of the center-of-mass of the particle. Examples of the results of the video tracking output are shown in Fig. 7 for two cases: no driving (blue) and high frequency stabilization (red). The trajectories have been superimposed on an image of the fast scanning laser spot to indicate the pendulum potential. For zero modulation amplitude the particle moves around the potential, in the direction shown by the arrow, to become localized in the region of *ϕ* = 0. For high—above threshold—driving amplitude, the colloidal particle stabilizes away from *ϕ* = 0 as shown, even in the presence of the uniform fluid drag force.

### Calibration and experimental parameters

**Figure 8:**
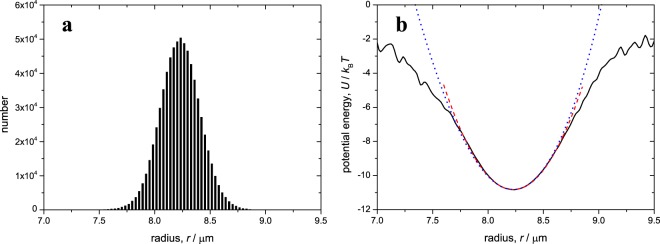
Measuring the optical potential parameters. (**a**) Histogram of fluctuations in the radial direction due to Brownian motion, as a result of *N* = 7.2 × 10^5^ samples of the radial position in a typical experiment. The corresponding average radius is $\langle r\rangle =L=8.228\pm 0.001\,\mu {\rm{m}}$. (**b**) Potential energy in the radial direction reconstructed from the data shown in the left panel (solid black line), together with a quadratic fit to potential minimum using *κ*_*r*_ = 0.125 p*Nμ*m^−1^ (dashed red line), and the associated quartic potential (dotted blue line).

The radial stiffness of the pendulum potential is calibrated by allowing the particle to diffuse freely in the optical potential with no fluid drag force nor driving vibration applied. An example histogram of *N* = 7.2 × 10^5^ samples of the radial position derived from DVM is shown in Fig. 8a. From the associated probability density *P*(*r*), the radius of the circular potential (length of the pendulum) is measured as $L=\langle r\rangle =8.228\pm \mathrm{0.001\ }\mu {\rm{m}}$. The pendulum potential energy in the radial direction is then reconstructed from the radial probability density as *U*(*r*)/*k*_*B*_*T*∝ − ln(*P*(*r*))^21,28^ as shown in Fig. 8b (solid black line). Near the minimum (*r* = *L*) the potential is approximately quadratic, $U(r)\approx U(L)+\frac{1}{2}{\kappa }_{r}{(r-L)}^{2}$ (dashed red line in Fig. 8b), with *κ*_*r*_ the radial spring constant, which from the fitted curve is *κ*_*r*_ = 0.125 ± 0.001 pN*μ*m^−1^. The pendulum proper frequency is then found from the radial spring constant as ${\omega }_{0}=\sqrt{{\kappa }_{r}/m}$.

In all experiments we used a *d* = 1.5 *μ*m diameter silica sphere, with mass *m* = *ρπd*^3^/6 = 3.53 × 10^−15^ kg, where *ρ* = 2.0 × 10^3^ kg/m^3^ is the mass density provided by the particle’s maker.

The dynamic viscosity of water at 25 °C, the approximate temperature of the experiments, is *ζ* = 0.89 mPa · s. Note that at 23 °C water viscosity is *ζ* = 0.94 mPa · s, which represents a relative uncertainty of about 5%—one of the largest sources of uncertainty in our experiments. The Stokes drag coefficient for the colloidal particle in water is then *η* = 3*πζd* = 1.26 × 10^−8^ Nsm^−1^, and the damping coefficient $\gamma =3\pi \zeta d/m\approx 2\pi \times 567\cdot {10}^{3}\,{\rm{H}}z$.

The experimental data of Fig. 4 correspond to *L* = 8.23 *μ*m. The set of filled points in Fig. 4 were obtained with an optical potential with *κ*_*r*_ = 0.125 pN*μ*m^−1^ (leading to *ω*_0_ = 2*π* × 947 Hz) and stage speed *v*_*s*_ = 1 *μ*ms^−1^ (leading to the pendulum’s natural frequency ${\rm{\Omega }}=\sqrt{\gamma {v}_{s}/L}=2\pi \times \mathrm{105\ }{\rm{Hz}}$), while the empty points in Fig. 4 correspond to *κ*_*r*_ = 0.310 pN*μ*m^−1^ (*ω*_0_ = 2*π* × 1490 Hz) and *v*_*s*_ = 0.76 *μ*ms ^−1^ (Ω = 2*π* × 91 H*z*). In both cases the modulation frequency was fixed to *ω* = 2*π* × 500 Hz.

The experimental results depicted in Fig. 5 were carried out with an optical pendulum with a slightly larger radius *L* = 8.611 *μ*m. The specific values of the optical trap stiffness and stage speed are shown in Table 1. The values of *γ*/*ω*_0_ are varying for each set, but larger than 200 in all four cases, thus also lying in the overdamped regime.

**Table 1:** Detailed data of the four sets shown in Fig. 5. They all share a common ratio *ω*_0_/Ω = 26.4 ± 0.1.

	*κ*_*r*_ (p*Nμ*m^−1^)	*v*_*s*_ (*μ*m*s*^−1^)
1	1.017	1.00
2	0.741	0.73
3	0.675	0.66
4	0.468	0.46

### The inverted pendulum at the micrometer scale

The stiffest optical pendulum we could obtain with the laser at maximum power corresponds to a proper frequency of about *ω*_0_ = 2*π* × 2700 Hz, which implies a ratio *γ*/*ω*_0_ = 210. This value is too high for the necessary condition for the inverted pendulum regime obtained above, i.e. $\gamma /{\omega }_{0} < 1$. However, given that air has a dynamic viscosity *ζ* which is about 50 times smaller than water, using larger colloidal particles and/or a more powerful laser could put the ratio *γ*/*ω*_0_ below unity, thus allowing the observation of the inverted pendulum regime at the micrometer scale with a similar experimental setup than the one proposed here. This is facilitated by the fact that aerosol optical tweezers are well established^30^ nowadays. The resulting demonstration of the inverted pendulum regime would add to the existing numerous experimental demonstrations of the inverted pendulum at the macroscopic scale.

## References

[1] Kapitza PL (1951). Dynamic stability of the pendulum with vibrating suspension point (in Russian). Sov. Phys JETP.

[2] Kapitza PL (1965). Collected papers of P. L. Kapitza.

[3] Stephenson A (1908). On an induced stability. Phil. Mag..

[4] Landa, P. S. *Nonlinear Oscillations and Waves in Dynamical System*s. Mathematics and Its Applications (Springer, 1996).

[5] Landau, D. L. & Lifshitz, E. M. *Mechanic*s (Pergamon, Oxford, 1976).

[6] Blekhman, I. I. *Vibrational Mechanics, Nonlinear Dynamics Effects, General Approach, Application*s (World Scientific Publishing Co. Pte. Ltd., Singapore, 2000).

[7] Landa PS, McClintock PVE (2000). Vibrational resonance. J. Phys. A.

[8] Bukov M, D’Alessio L, Polkovnikov A (2015). Universal high-frequency behavior of periodically driven systems: from dynamical stabilization to floquet engineering. Adv. in Phys..

[9] Borromeo M, Marchesoni F (2007). Artificial sieves for quasimassless particles. Phys. Rev. Lett..

[10] Bagnato VS, Bigelow NP, Surdutovich GI, Zilio S (1994). Dynamical stabilization: a new model for supermolasses. Optics Lett..

[11] Grifoni M, Hänggi P (1995). Coherent and incoherent quantum stochastic resonance. Phys. Rev. Lett..

[12] Aidelsburger M (2014). Measuring the chern number of hofstadter bands with ultracold bosonic atoms. Nat. Phys..

[13] Wickenbrock A (2012). Vibrational mechanics in an optical lattice: Controlling transport via potential renormalization. Phys. Rev. Lett..

[14] Chizhevsky VN, Smeu E, Giacomelli G (2003). Experimental evidence of vibrational resonance in an optical system. Phys. Rev. Lett..

[15] Chizhevsky VN (2014). Experimental evidence of vibrational resonance in a multistable system. Phys. Rev. E.

[16] Casado-Pascual J, Cubero D, Baltanas J (2007). Stochastic resonance with weak monochromatic driving: Gains above unity induced by high-frequency signals. EPL.

[17] Cubero D, Baltanas JP, Casado-Pascual J (2006). High-frequency effects in the Fitzhugh-Nagumo neuron model. Phys. Rev. E.

[18] Bordet M, Morfu S (2013). Experimental and numerical study of noise effects in a fitzhugh–nagumo system driven by a biharmonic signal. Chaos, Solitons Fractals.

[19] Weinberg SH (2014). High frequency stimulation of cardiac myocytes: A theoretical and computational study. Chaos.

[20] Uzuntarla M, Yilmaz E, Wagemakers A, Ozer M (2015). Vibrational resonance in a heterogeneous scale free network of neurons. Commun. Nonlinear Sci. Numer. Simulat..

[21] Cubero, D. & Renzoni, F. *Brownian ratchets: From Statistical Physics to Bio and Nano-motor*s (Cambridge University Press, Cambridge, 2016).

[22] Maragò OM, Jones PH, Gucciardi PG, Volpe G, Ferrari AC (2013). Optical trapping and manipulation of nanostructures. Nat. Nanotechnology.

[23] Bender, C. M. & Orszag, S. A. *Avanced mathematical methods for scientist and engineer*s (McGraw-Hill, New York, 1978).

[24] Casado-Pascual J (2010). Effect of a high-frequency magnetic field on the resonant behavior displayed by a spin-1/2 particle under the influence of a rotating magnetic field. Chem. Phys..

[25] Blackburn JA, Smith HJT, Gronbech-Jensen N (1992). Stability and Hopf bifurcations in an inverted pendulum. Am. J. Phys..

[26] Bartuccelli MV, Gentile G, Georgiou KV (2001). On the dynamics of a vertically driven damped planar pendulum. Proc. R. Soc. Lond. A.

[27] Carbo RM, Smith RWM, Poese ME (2010). Stability of the parametrically excited damped inverted pendulum: Theory and experiment. J. Acoust. Soc. Am..

[28] Jones, P. H., Maragò, O. M. & Volpe, G. *Optical Tweezers: Principles and Applications* (Cambridge University Press, Cambridge, 2015).

[29] Jones PH, Maragò OM, Stride EPJ (2007). Parametrization of trapping forces on microbubbles in scanning optical tweezers. J. Opt. A: Pure Appl. Opt.

[30] Omori R, Kobayashi T, Suzuki A (1997). Observation of a single-beam gradient-force optical trap for dielectric particles in air. Opt. Lett..

[31] MacDonald MP, Spalding GC, Dholakia K (2003). Microfluidic sorting in an optical lattice. Nature.

[32] Chou CF (1999). Sorting by diffusion: An asymmetric obstacle course for continuous molecular separation. Proc. Natl. Acad. Sci. USA.

[33] van Oudenaarden A, Boxer SG (1999). Brownian ratchets: Molecular separations in lipid bilayers supported on patterned arrays. Science.

[34] Matthias S, Müller F (2003). Asymmetric pores in a silicon membrane acting as massively parallel brownian ratchets. Nature.

